# Clinical effects of laser-based cavity preparation on class V resin-composite fillings

**DOI:** 10.1371/journal.pone.0270312

**Published:** 2022-06-23

**Authors:** Markus Heyder, Bernd Sigusch, Christoph Hoder-Przyrembel, Juliane Schuetze, Stefan Kranz, Markus Reise

**Affiliations:** 1 Department of Conservative Dentistry and Periodontology, University Hospital, Jena, Germany; 2 Department of Fundamental Science, University of Applied Sciences, Jena, Germany; Virginia Commonwealth University, UNITED STATES

## Abstract

The aim of the present clinically controlled two-year study was to investigate the influence of laser-based cavity preparation on the long-term performance of Class V resin-composite fillings. Class V non-carious lesions (n = 75) were randomly assigned to two test and one control group. Cavities in both test groups were prepared using an Er,Cr:YSGG laser (Waterlase MD, Biolase, Irvine, California, USA). The device was operated at 3 W (150 mJ, 30 J/cm^2^), 50% water, 60% air, 30 Hz in H mode. Subsequently, laser-prepared tooth surfaces in test group I (n = 21) were additionally conditioned by acid etching (etch-and-rinse). Laser-prepared cavities of test group II (n = 21) received no additional acid conditioning. After application of an adhesive, all cavities were restored using the resin-composite Venus®. For cavities in the control group (n = 33) conventional diamond burs were used for preparation which was followed by an etch-and-rinse step, too. The fillings were evaluated immediately (baseline) and after 6, 12 and 24 months of wear according to the C-criteria of the USPHS-compatible CPM-index. The results showed that after 24 month of wear, laser-preparation was associated with fillings of high clinical acceptability. Compared to conventional bur-based treatment, laser-based cavity preparation resulted in fillings with high marginal integrity and superior marginal ledge configurations (p = 0.003). Furthermore, laser-preparation combined with additional acid-conditioning (test group I) resulted in fillings with the best marginal integrity and the lowest number in marginal discoloration, especially at the enamel-composite margins (p = 0.044). In addition, total loss of fillings was also less frequently observed in both laser groups as compared to the control. The results clearly demonstrate that laser-based cavity preparation will benefit the clinical long-time performance of Class V resin-composite fillings. Furthermore, additional acid-conditioning after laser preparation is of advantage.

## Introduction

In modern day dental practice, the use of lasers is common and often seen as a favourable alternative to conventional treatment methods [1–7].

Current dental ablative laser systems are based on innovative techniques which enable efficient and secure minimal-invasive hard-tissue preparation [8–11]. Especially in case of Class V lesions, laser-based cavity preparation already showed favourable characteristics *In-vitro* [12].

In restorative dentistry, nano-filled resin-composites are preferably used, especially because of their high aesthetic appearance and sufficient clinical performance [13–15]. Nevertheless, of all resin-based composite restorations, Class V fillings are still afflicted by the lowest longevity [16]. Clinically, this appears in the formation of marginal gaps, discolorations, increased microleakage, postoperative hypersensitivity, loss of retention and secondary caries [17–21]. In addition, occlusal stress shielding and the high degree of dentin sclerosis, especially found among non-carious cervical lesions, are further reasons for Class V filling failure [22].

Thus, to increase retention and the micro-tensile bonding strength, mechanical cavity preparation is recommended prior to any restorative measure [23, 24].

In this context, it was shown that especially in non-carious lesions, laser-based cavity preparation has a positive effect upon the bonding strength of resin-composite fillings [12]. In comparison to traditional bur-based treatment procedures, laser preparation leads to the formation of specific micromorphologic surface patterns which are associated with improved bonding characteristics [25]. In detail, laser treated surfaces are characterized by exposed enamel prisms, wide opened dentinal tubes and the absence of a smear layer [26–29].

As already proven by Galafassi et al. in a 12-month clinical trial, laser-based cavity preparation has a positive effect upon the performance of Class I composite restorations [30]. Furthermore, Er:YAG laser-prepared Class V fillings revealed a more sufficient marginal seal on occlusal and gingival margins as compared to conventional bur-cut restorations *In-vitro* [31].

To date, clinical studies evaluating the long-term outcome of laser-preparation especially in Class V resin-composite restorations are still rare [32].

Therefore, the present clinical trial aimed at investigating the performance of Class V fillings with cavities prepared using an Er,Cr:YSGG solid-state laser in non-carious cervical lesions. The results were compared to the outcome of Class V fillings placed in traditional bur-cut cavities. Since laser-treated tooth surfaces are of rough appearance the need of an additional acid conditioning is still controversially discussed [32, 33]. In this regard, the present study also observed the effect of an additional etch-and-rinse approach on the clinical long-term success of laser-prepared Class V composite restorations, too.

## Materials and methods

### Patient recruitment

**Fig 1:**
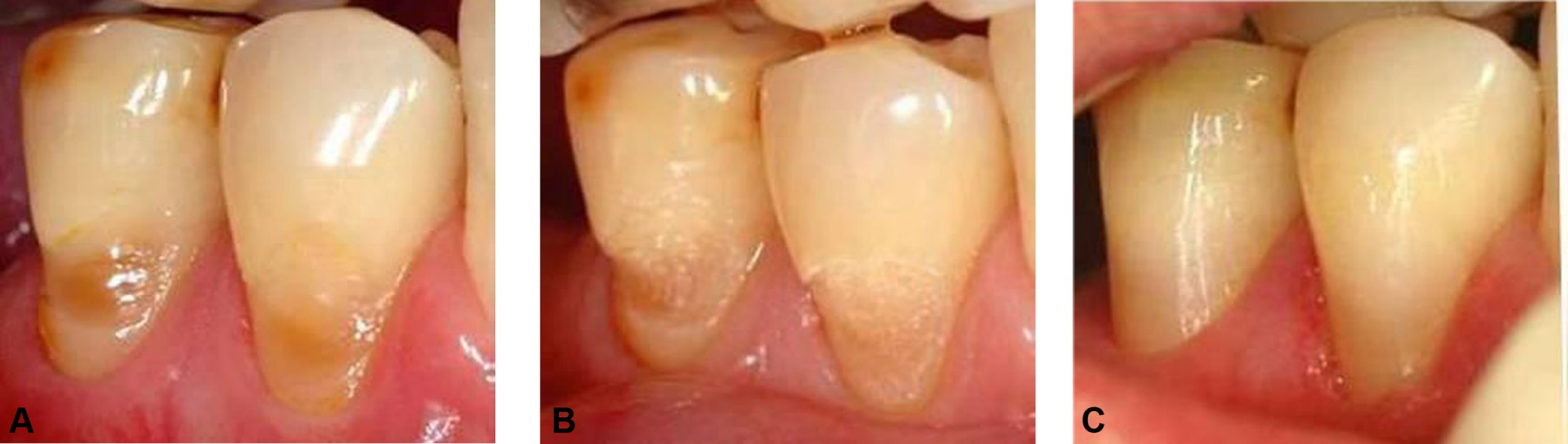
Non-carious cervical lesions of teeth 44 and 45 after laser preparation and restoration with Venus® (Heraeus Kulzer, Germany). Class V resin-composite restauration of a non-carious cervical lesions in premolars (44, 45). (A) Initial lesion. (B) After Er,Cr:YSGG laser-based cavity preparation. (C) Cured and finished resin-composite restauration.

The present study involved a total of 29 patients recruited from the Department of Conservative Dentistry and Periodontology, Jena University Hospital, Germany. Each patient showed at least one non-carious defect in the cervical region with exposed dentin (Fig 1A). Defects in molars were not included in the study. All participants had to be at least 18 years of age. The age distribution ranged in between 22 and 89 years with a mean age of 56.24 years. The study has been approved by the Ethical Committee of the Friedrich Schiller University, Jena, Germany (No. 2013-05/07) and written informed consent of each patient was given prior to any therapy.

Teeth with cervical lesions due to caries and teeth intended for denture prosthetics in the nearby future were excluded from the study. Other reasons for exclusion were (i) insufficient oral hygiene (approximal plaque index and bleeding on probing > 30%), (ii) excessive smoking (> 20 cigarettes per day), and/or (iii) the consumption of > 5 cups of coffee or black tea per day.

Within this study, a total of 75 Class V lesions were included. After randomization, the patients were assigned into two test groups and one control group (Table 1):

Test group I: laser cavity preparation *followed by* etch-and-rinse (L + e&r), n = 21,
Test group II: laser cavity preparation *without* etch-and-rinse (L–e&r), n = 21,
Control group: cavity preparation using conventional diamond burs, n = 33.

**Table 1:** Experimental group set-up.

group	Abbreviation	Description	number of fillings at baseline
Test group I	L + e&r	laser preparation with etch-and-rinse	n = 21
Test group II	L—e&r	laser preparation without etch-and-rinse	n = 21
Control group	control	conventional cavity preparation	n = 33

Randomized assignment of the fillings to the test and control groups.

### Laser-based cavity preparation

In both test groups laser-based preparation was carried out using an **Er,Cr:YSGG** laser (Waterlase MD, Biolase, Irvine, California, USA). The device was operated at 3 W (150 mJ, 30 J/cm^2^), 50% water, 60% air, 30 Hz in H mode. For preparation, a “MG6“sapphire tip with a diameter of 600 μm and a length of 6 mm was used and guided across the tooth surface at distances of 1–1.5 mm, until a laser-typical roughened surface of whitish-opaque appearance was received (Fig 1B). An air/water flow rate of 33 ml/min was applied.

### Bur-based cavity preparation

In the control group, conventional bure-based preparation was carried out, following the rules of retentive preparation with bevelled enamel edges. For this purpose, a cylindrical 107 μm diamond bur was used (Komet, Gebr. Brasseler GmbH & Co. KG, Lemgo, Germany) in a contra-angle handpiece 1:5 under constant water cooling (KaVo Dental, Biberach/Riß, Germany). In the marginal region adjacent to the enamel an additional bevel of at least 1 mm of width was prepared, using a flame-shaped 46 μm fine-grain diamond bur (Komet, Gebr. Brasseler GmbH & Co. KG, Lemgo, Germany).

### Composite application

Prior to the placement of a restauration, the cervical margin was exposed by means of a retraction cord (Ultrapak®, #0 and #00; Ultradent, South Jordan, USA).

In test group I and in the control group, total etching was performed by applying 37% orthophosphoric acid gel for 15 s on dentin and 30 s on enamel (Total Etch™, Ivoclar Vivadent AG, Schaan, Liechtenstein).

Subsequently, all cavities were treated with Gluma® Solid Bond P and Gluma® Solid Bond S (Heraeus Kulzer GmbH, Hanau, Germany) as advised by the manufacturer followed by light curing for 40 s each (Translux® PowerBlue®, Heraeus Kulzer GmbH, Hanau, Germany).

Subsequently, the cavities were filled with the resin-composite Venus® (Heraeus Kulzer GmbH, Hanau, Germany), which was applied in 2 mm thick layers, with each being light-cured for 40 s. For finishing the restoration flame-shaped fine and extra fine diamond finishing burs (Komet, Gebr. Brasseler GmbH & Co. KG, Lemgo, Germany; red ring 46 μm, yellow ring 25 μm) were used. The final finish was applied using silicone polishers (Busch & Co. KG, Engelskirchen, Germany, yellow, blue) (Fig 1C).

### Clinical evaluation

All fillings were evaluated by a trained and blinded independent professional using a 2.5x magnification head-worn loupe (EYEMAG Smart sports, Bajohr, Carl Zeiss Meditec AG, Jena, Germany).

Tactile and visual clinical evaluation of the fillings was carried out immediately after application (baseline) and after 6, 12, and 24 months of wear, with the aid of a mirror and a fine dental probe (# 3A, Hu-Friedy Mfg. Co., Inc., Chicago, Illinois, USA).

The qualitative assessment of each filling was performed according to the C-criteria of the US Public Health Service compatible CPM index [13, 14, 34–36]. In brief, the C-criteria comprise a clinical evaluation of the anatomical form, marginal integrity, marginal ledge condition, degree of marginal discoloration and the overall clinical acceptance by 4 different grades with ‘code 0’ best and ‘code 3’ worst value. All criteria were assessed separately for the enamel-composite and the dentine-composite filling margins.

### Statistical analysis

Statistical data analysis was performed using SPSS® version 19 for Windows (SPSS Inc., Chicago, IL, USA).

Changes to all restorations starting at baseline up to the final inspection were examined by means of the Friedman test. The Kruskal-Wallis test was used for the global statistical comparison of the methods. Significance between and within the groups was tested by applying the Mann-Whitney U-test.

The level of significance was set to p < 0.05.

## Results

During the study period, 53 cervical restorations out of 75 fillings placed at baseline (70,7%) could be evaluated throughout the entire observation time, while 22 fillings could not be re-evaluated because of total (n = 8) or partial loss (n = 12), or because patients did not show up to the recall appointment (n = 2).

As shown in Table 2, all 75 fillings displayed correct anatomical forms at baseline (Code 0). Throughout the 2-year examination period, partial changes in the filling contour (Code 1) were detected among all groups.

**Table 2:** Results evaluated according to the C-criteria “anatomical form”.

C-criteria "anatomical form"						
			code 0	code 1	code 2	code 3
examination time	group	number of fillings examined	clinical correct filling	lack of filling contour	partial loss of filling	total loss of filling
baseline	test group 1	n = 21	21 (100.0%)	-	-	-
	test group 2	n = 21	21 (100.0%)	-	-	-
	control	n = 33	33 (100.0%)	-	-	-
6 months	test group 1	n = 21	16 (76.2%)	2 (9.5%)	3 (14.3%)	-
	test group 2	n = 18	18 (100.0%)	-	-	-
	control	n = 33	28 (84.4%)	-	1 (3.0%)	4 (12.1%)
12 months	test group 1	n = 16	15 (93.8%)	-	-	1 (6.2%)
	test group 2	n = 18	15 (83.3%)	-	3 (16.7%)	-
	control	n = 28	23 (82.1%)	1 (3.6%)	2 (7.1%)	2 (7.1%)
24 months	test group 1	n = 15	13 (86.7%)	-	2 (13.3%)	-
	test group 2	n = 15	15 (100.0%)	-	-	-
	control	n = 23	21 (91.3%)	-	1 (4.3%)	1 (4.3%)

Clinical assessment of the fillings by the C-criteria “anatomical form”.

Total loss of fillings (Code 3) occurred most frequently within the control group. In detail, after 6 months of wear 4 restorations were lost completely, followed by another two fillings after 12 months and one after 24 months.

In test group I only one filling was lost completely (Code 3) after 12 months of wear, whereas in test group II none of the placed restorations were entirely lost during the 24-month study period.

Besides that, partial lost (Code 2) was documented within the control group at all examination points (n = 4), while in test group I partial lost occurred after 6 and 24 months of wear (n = 5) and in test group II after 12 months (n = 3), only.

All teeth remained vital throughout the entire examination period.

For the criteria “anatomical form”, no significant differences were determined among all evaluation points.

The integrity of the filling margins was examined separately for the enamel and dentin margins (Table 3). At all examination times, fillings in the test groups showed less insufficient margins adjacent to the enamel (Code 1 and 2) as compared to the control. After one year this result turned up to be significant (p = 0.003). Between both test-groups no significant difference was observed (p = 0.513).

**Table 3:** Evaluation of Class V restorations according to the C-criteria “marginal integrity”, “marginal ledge”, “marginal discoloration” of the CPM index.

**C-criteria "marginal integrity"**										
			enamel/composite margin				dentin/composite margin			
			code 0	code 1	code 2	code 3	code 0	code 1	code 2	code 3
examination time	group	number of fillings examined	perfect margin	up to 1/3 of the circumference can be probed	1/3 to 2/3 of the circumference can be probed	marginal leakage	perfect margin	up to 1/3 of the circumference can be probed	1/3 to 2/3 of the circumference can be probed	marginal leakage
baseline	test group 1	n = 21	21 (100.0%)	-	-	-	21 (100.0%)	-	-	-
	test group 2	n = 21	21 (100.0%)	-	-	-	21 (100.0%)	-	-	-
	control	n = 33	33 (100.0%)	-	-	-	33 (100.0%)	-	-	-
6 months	test group 1	n = 17	15 (88.2%)	1 (5.9%)	1 (5.9%)	-	14 (82.4%)	1 (5.9%)	2 (11.8%)	-
	test group 2	n = 18	14 (77.8%)	3 (16.7%)	1 (5.6%)	-	15 (83.3%)	3 (16.7%)	-	-
	control	n = 28	14 (50.0%)	11 (39.3%)	3 (10.7%)	-	21 (75%)	6 (21.4%)	1 (3.6%)	-
12 months	test group 1	n = 15	11 (73.3%)	4 (26.7%)	-	-	11 (73.7%)	3 (20.0%)	1 (6.7%)	-
	test group 2	n = 16	10 (62.5%)	4 (25.0%)	2 (12.5%)	-	9 (56.3%)	5 (31.3%)	2 (12.5%)	-
	control	n = 24	5 (20.8%)	13 (54.2%)	6 (25.0%)	-	14 (58.3%)	6 (25.0%)	4 (16.7%)	-
24 months	test group 1	n = 14	8 (57.1%)	2 (14.3%)	4 (28.6%)	-	10 (71.4%)	2 (14.3%)	2 (14.3%)	-
	test group 2	n = 15	7 (46.7%)	7 (46.7%)	1 (6.7%)	-	8 (53.3%)	5 (33.3%)	1 (6.7%)	1 (6,7%)
	control	n = 21	3 (14.3%)	10 (47.6%)	8 (38.1%)	-	16 (76.2%)	3 (14.3%)	2 (9.5%)	-
**C-criteria "marginal ledge"**										
			enamel/composite margin				dentin/composite margin			
			code 0	code 1	code 2	code 3	code 0	code 1	code 2	code 3
examination time	group	number of fillings examined	no ledge	positive ledge	negative ledge	positive and negative ledge	no ledge	positive ledge	negative ledge	positive and negative ledge
baseline	test group 1	n = 21	21 (100.0%)	-	-	-	100.0	-	-	-
	test group 2	n = 21	21 (100.0%)	-	-	-	100.0	-	-	-
	control	n = 33	33 (100.0%)	-	-	-	100.0 ,0	-	-	-
6 months	test group 1	n = 18	15 (83.3%)	1 (5.6%)	2 (11.1%)	-	14 (77.8%)	-	4 (22.2%)	-
	test group 2	n = 18	14 (77.8%)	4 (22.2%)	-	-	15 (83.3%)	2 (11.1%)	1 (5.6%)	-
	control	n = 28	15 (53.6%)	12 (42.9%)	1 (3.6%)	-	21 (75.0%)	4 (14.3%)	3 (10.7%)	-
12 months	test group 1	n = 15	10 (66.7%)	4 (26.7%)	1 (6.7%)	-	11 (73.3%)	-	4 (26.7%)	-
	test group 2	n = 16	10 (62.5%)	6 (37.5%)	-	-	9 (56.3%)	4 (25.0%)	2 (12.5%)	1 (6.3%)
	control	n = 24	5 (20.8%)	15 (62.5%)	4 (16.7%)	-	14 (58.4%)	2 (8.3%)	6 (25.0%)	2 (8.3%)
24 months	test group 1	n = 14	8 (57.1%)	5 (35.7%)	1 (7.1%)	-	9 (64.3%)	-	5 (35.7%)	-
	test group 2	n = 15	7 (46.7%)	7 (46.7%)	1 (6.7%)	-	8 (53.3%)	3 (20.0%)	4 (26.7%)	-
	control	n = 21	3 (14.3%)	14 (66.7%)	3 (14.3%)	1 (4.8%)	16 (76.2%)	2 (9.5%)	3 (14.3%)	-
**C-criteria "marginal discoloration"**										
			enamel/composite margin				dentin/composite margin			
			code 0	code 1	code 2	code 3	code 0	code 1	code 2	code 3
examination time	group	number of fillings examined	no marginal discoloration	discoloration on up to 1/3 of the circumference	discoloration on more than 1/3 of the circumference	secondary caries with cavitation	no marginal discoloration	Discoloration on up to 1/3 of the circumference	discoloration on more than 1/3 of the circumference	secondary caries with cavitation
baseline	test group 1	n = 21	21 (100.0%)	-	-	-	21 (100.0%)	-	-	-
	test group 2	n = 21	21 (100.0%)	-	-	-	21 (100.0%)	-	-	-
	control	n = 33	33 (100.0%)	-	-	-	33 (100.0%)	-	-	-
6 months	test group 1	n = 18	17 (94.4%)	1 (5.6%)	-	-	18 (100.0%)	-	-	-
	test group 2	n = 18	15 (83.3%)	3 (16.7%)	-	-	18 (100.0%)	-	-	-
	control	n = 29	21 (72.7%)	8 (27.6%)	-	-	29 (100.0%)	-	-	-
12 months	test group 1	n = 15	13 (86.7%)	1 (6.7%)	1 (6.7%)	-	14 (93.3%)	1 (6.7%)	-	-
	test group 2	n = 16	11 (68.8%)	5 (31.3%)	-	-	14 (87.5%)	1 (6.3%)	1 (6.3%)	-
	control	n = 24	14 (58.3%)	8 (33.3%)	2 (8.3%)	-	23 (95.8%)	1 (4.2%)	-	-
24 months	test group 1	n = 14	11 (78.6%)	1 (7.1%)	2 (14.3%)	-	12 (85.7%)	2 (14.3%)	-	-
	test group 2	n = 15	5 (33.3%)	10 (66.7%)	-	-	12 (80.0%)	1 (6.7%)	2 (13.3%)	-
	control	n = 22	11 (50.0%)	8 (36.4%)	3 (13.6%)	-	21 (95.5%)	1 (4.5%)	-	-

Results of the examined C-criteria (marginal integrity, marginal ledge formation, degree of marginal discoloration) according to the CPM-Index assessed for the composite-enamel and composite-dentin margins.

At the composite-dentin margins similar results were obtained. After one year of wear in test group I filling margins in the region adjacent to the dentin were explorable at a distinctly lower rate compared to the control group. After 24 months of wear, only one filling in test group II was rated with code 3 in the region adjacent to the dentin. Overall, in regard to the criteria “marginal integrity” no significance differences were detected at all evaluation points. The obtained p-values ranged in between p = 0.797 for 6 month and p = 0.438 for 12 month.

Similar results were documented for the marginal ledge assessment. Here again, in both test groups fewer ledge formation in the region adjacent to the enamel were observed compared to the control group. These results turned out to be significant after one year of wear (p = 0.003). In test group I most of the fillings were not afflicted by marginal ledge formation (Code 0). Whereas among the control-group positive ledges were more frequently detected.

Insufficient marginal ledge formation adjacent to dentin (apical region) were also less frequently observed in both test groups as compared to the control. Notably, in all groups the number of insufficient marginal ledges increased by time (Table 3).

Discoloration of the filling margins were detected among all groups most frequently in the region adjacent to the enamel. After two years of wear, fillings in test group I showed the lowest rates in discolorations (Code 1 and 2) compared to test group II and the control group, which was rated to be significant (p = 0.044).

**Fig 2:**
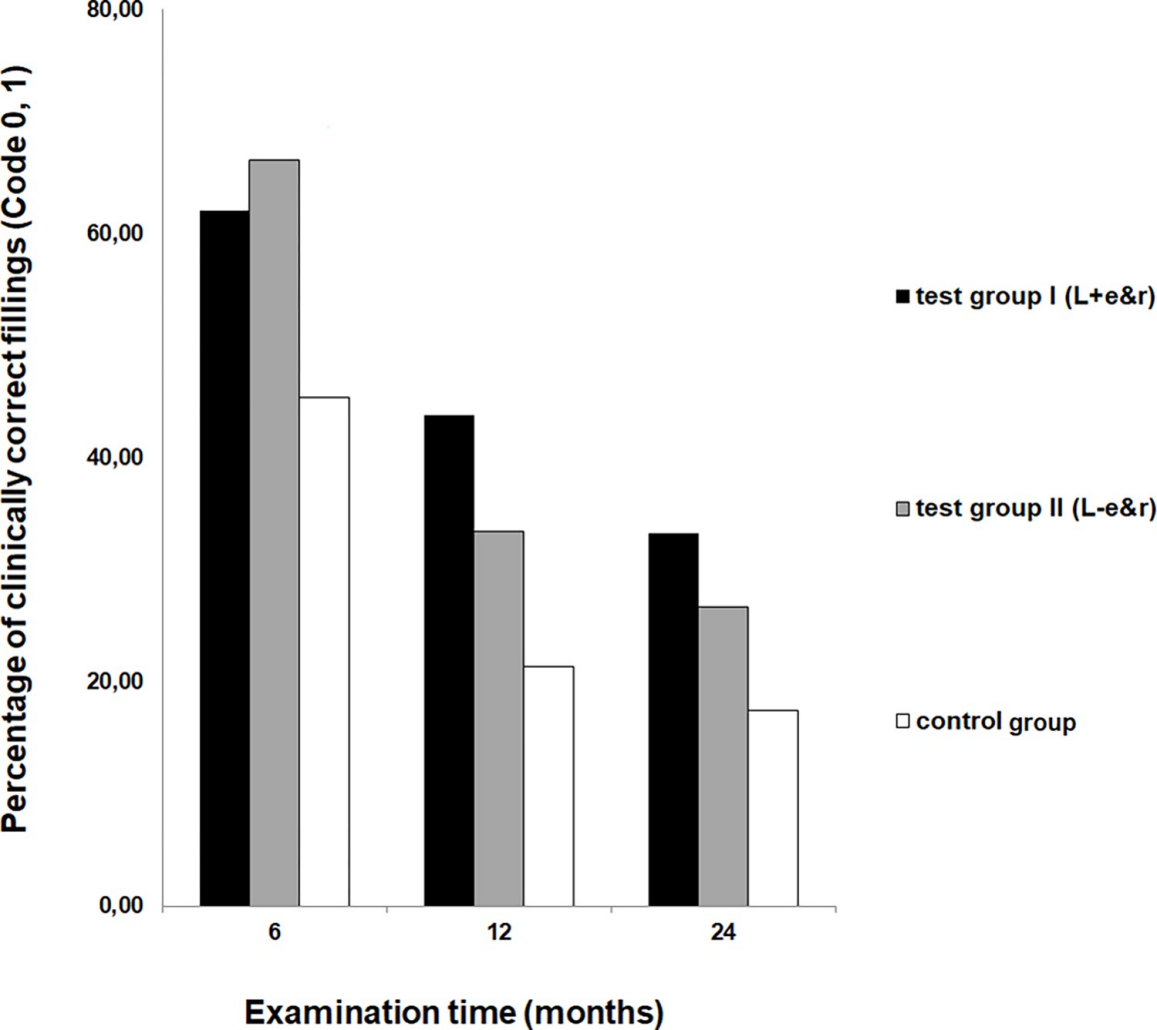
Clinical acceptance. The diagram shows the results assessed for the C-criteria “clinical acceptance” for all groups after 6, 12 and 24 months of wear.

In summary, in both laser test groups the highest number in clinically acceptable fillings were detected after 6, 12 and 24 months of wear (Fig 2).

## Discussion

The present *In-vivo-*study clearly demonstrates, that laser-based cavity preparation has a positive impact on the long-term performance of Class V resin-composite restorations. Moreover, fillings placed in laser-prepared cavities that were additionally conditioned with phosphoric acid showed the best clinical results.

In the present study an Er,Cr:YSGG laser operated at 3 W (150 mJ, 30 J/cm^2^), 50% water, 60% air, 30 Hz in H mode was used for preparation, equipped with a “MG6“sapphire tip of 600 μm in diameter and 6 mm in length. The sapphire tip was guided in a distance of app. 1 mm over the tooth surface until a rough surface of whitish-opaque appearance was received.

As already observed by various authors, laser based preparation leads to dentin and enamel surfaces with superior bonding characteristics [12, 29, 37].

Microscopic studies have revealed that laser-ablation results in the formation of specific irregular micro-retentive surface patterns that are characterized by exposed enamel prisms, wide opened dentinal tubes and the absence of a smear layer [11, 26–29, 38]. Further, it was shown that in comparison to bur-cut surfaces, a more sufficient hybrid layer with a pronounced formation of resin tags can be established on laser-prepared dentin surfaces [39, 40]. In this context, the mentioned characteristics might probably be seen as reasons for the superior performance of laser-prepared fillings in the present study.

The clinical performance of the placed resin-composite restorations was evaluated according to the C-criteria of the US Public Health Service compatible CPM-index. The index enables a standardized clinical assessment which includes an evaluation of the anatomical form, marginal integrity, marginal ledge configuration, degree of marginal discoloration and overall clinical acceptance [13, 14, 34–36].

In detail, as shown in the present clinical study, after one year of wear excellent results were documented especially for the criteria ‘marginal integrity’ with the highest values examined for the enamel-composite margins. In this context it was proven in a recent *In-vivo* study of our group that especially the integrity of the filling margin has a strong effect upon the survival rate of resin-composite fillings [14].

Furthermore, the present study revealed that insufficient ledge formation was less frequently detected among fillings placed in laser-cut cavities, too. The occurrence of positive and also negative ledges was observed to a higher extent in the control group in which conventional burs were used for preparation. Similar to these results, previous findings of our group showed that negative marginal ledges are formed to a much higher rate within the first period of wear [13, 14].

In regard to the criteria “marginal discoloration” the present study showed, that discolorations especially at the composite enamel margins were found to a significant higher rate in the control group as compared to the laser groups. Further, throughout the entire study period the highest number in fillings without discolorations were detected in test group I (laser preparation in combination with etch-and-rinse).

The results are in line with previous findings obtained from *In-vitro* studies which confirm that compared to bur-based treatment, laser preparation is associated with improved bonding properties and less marginal discoloration [41, 42].

Overall, in the present study the highest number in clinically correct fillings was detected among both laser groups. Similar results were described by Hamidi et al. who also reported on superior effects of laser-based cavity preparation *In-vivo* [43].

Besides filling preparation, ablative dental lasers can be used in other treatment fields, such as caries removal or endodontic therapy, too [17, 22, 44–46]. But, especially in case of non-carious cervical lesions, laser preparation can be seen as a preferable alternative to conventional bur-based treatment procedures.

The present study showed that conventional preparation was associated with high numbers in fillings that were completely lost. In detail, only one filling was lost completely in test group I, whereas 7 fillings were totally lost in the bur-treated control group. In addition, none of the restorations placed at baseline were lost completely in test group II. It is interesting to mention, that Preussker et al. reported similar results for Class V filling after bur-based preparation [47].

However, there are still controversial discussions about the efficiency of laser-preparation and the right choice of adhesive systems used in combination with or without acid pre-treatment [32, 33, 48]. Since laser treatment leads to rough and uneven surfaces without smear layer, application of self-etch adhesive systems is advised. Nevertheless, it seems to be of advantage when laser-cut tooth surfaces are additionally conditioned with phosphoric acid [32]. In this regard, Kiryk et al. clearly showed that Er:YAG laser-based preparation in combination with conventional acid conditioning results in improved adhesion properties [27].

As witnessed in the present study, too, pre-treatment with 37% orthophosphoric acid resulted in superior performance rates. Both, after one and two years of wear, the share of clinically correct fillings was higher in test group I (laser-preparation followed by acid-conditioning) as compared to test group II (laser-preparation without etch-and-rinse).

Best results were especially obtained for the enamel-composite margins. In this regard, it becomes obvious that laser-preparation followed by acid etching is associated with the best bonding effects. According to the classification of Silverstone, type I etching pattern are afflicted with the most superior adhesive properties [49, 50]. As a result of Er:YAG laser conditioning, similar structures compared to Silverstone’s type III patterns are received which are characterized by enamel prisms with damaged peripheral and central regions [27, 51, 52].

The results of the present study are in line with other authors that also reported on improved bonding properties when orthophosphoric acid was used to pre-treat laser prepared tooth surfaces [33, 53–55].

The present study concludes that laser-preparation in combination with an additional step of acid-etching will clearly improve the long-term performance of Class V resin-composite restorations.

## Conclusions

The present clinical study reveals, that Class V composite fillings placed in non-carious lesions after Er,Cr;YSGG laser-based cavity preparation shows significantly better clinical long-term results as compared to restorations that are associated with conventional burr-based cavity preparation. Moreover, surface pre-conditioning with an etching agent after laser preparation will further improve the clinical performance.

## Supporting information

S1 Dataset (DOCX)
