# Supplementary material for: Clinical effects of laser-based cavity preparation on class V resin-composite fillings
Source: PLoS One. 2022 Jun 23;17(6):e0270312. doi: 10.1371/journal.pone.0270312 (PMC9223344; doi:10.1371/journal.pone.0270312)
Supplement: S1 Dataset — (DOCX) [file pone.0270312.s001.docx]

**Minimal underlying data set**

| **C-criteria "anatomical form"** | | | | | | |
| --- | --- | --- | --- | --- | --- | --- |
|  | | | code 0 | code 1 | code 2 | code 3 |
| examination time | group | number of fillings examined | clinical correct filling | lack of filling contour | partial loss of filling | total loss of filling |
| baseline | test group 1 | n=21 | 21 (100.0%) | - | - | - |
|  | test group 2 | n=21 | 21 (100.0%) | - | - | - |
|  | control | n=33 | 33 (100.0%) | - | - | - |
| 6 months | test group 1 | n=21 | 16 (76.2%) | 2 (9.5%) | 3 (14.3%) | - |
|  | test group 2 | n=18 | 18 (100.0%) | - | - | - |
|  | control | n=33 | 28 (84.4%) | - | 1 (3.0%) | 4 (12.1%) |
| 12 months | test group 1 | n=16 | 15 (93.8%) | - | - | 1 (6.2%) |
|  | test group 2 | n=18 | 15 (83.3%) | - | 3 (16.7%) | - |
|  | control | n=28 | 23 (82.1%) | 1 (3.6%) | 2 (7.1%) | 2 (7.1%) |
| 24 months | test group 1 | n=15 | 13 (86.7%) | - | 2 (13.3%) | - |
|  | test group 2 | n=15 | 15 (100.0%) | - | - | - |
|  | control | n=23 | 21 (91.3%) | - | 1 (4.3%) | 1 (4.3%) |

| **C-criteria "marginal integrity"** | | | | | | | | | | |
| --- | --- | --- | --- | --- | --- | --- | --- | --- | --- | --- |
|  | | | enamel/composite margin | | | | dentin/composite margin | | | |
|  |  |  | code 0 | code 1 | code 2 | code 3 | code 0 | code 1 | code 2 | code 3 |
| examination time | group | number of fillings examined | perfect margin | up to 1/3 of  the circumference can be probed | 1/3 to 2/3 of  the circumference can be probed | marginal leakage | perfect margin | up to 1/3 of  the circumference can be probed | 1/3 to 2/3 of  the circumference can be probed | marginal leakage |
| baseline | test group 1 | n=21 | 21 (100.0%) | - | - | - | 21 (100.0%) | - | - | - |
|  | test group 2 | n=21 | 21 (100.0%) | - | - | - | 21 (100.0%) | - | - | - |
|  | control | n=33 | 33 (100.0%) | - | - | - | 33 (100.0%) | - | - | - |
| 6 months | test group 1 | n=17 | 15 (88.2%) | 1 (5.9%) | 1 (5.9%) | - | 14 (82.4%) | 1 (5.9%) | 2 (11.8%) | - |
|  | test group 2 | n=18 | 14 (77.8%) | 3 (16.7%) | 1 (5.6%) | - | 15 (83.3%) | 3 (16.7%) | - | - |
|  | control | n=28 | 14 (50.0%) | 11 (39.3%) | 3 (10.7%) | - | 21 (75%) | 6 (21.4%) | 1 (3.6%) | - |
| 12 months | test group 1 | n=15 | 11 (73.3%) | 4 (26.7%) | - | - | 11 (73.7%) | 3 (20.0%) | 1 (6.7%) | - |
|  | test group 2 | n=16 | 10 (62.5%) | 4 (25.0%) | 2 (12.5%) | - | 9 (56.3%) | 5 (31.3%) | 2 (12.5%) | - |
|  | control | n=24 | 5 (20.8%) | 13 (54.2%) | 6 (25.0%) | - | 14 (58.3%) | 6 (25.0%) | 4 (16.7%) | - |
| 24 months | test group 1 | n=14 | 8 (57.1%) | 2 (14.3%) | 4 (28.6%) | - | 10 (71.4%) | 2 (14.3%) | 2 (14.3%) | - |
|  | test group 2 | n=15 | 7 (46.7%) | 7 (46.7%) | 1 (6.7%) | - | 8 (53.3%) | 5 (33.3%) | 1 (6.7%) | 1 (6,7%) |
|  | control | n=21 | 3 (14.3%) | 10 (47.6%) | 8 (38.1%) | - | 16 (76.2%) | 3 (14.3%) | 2 (9.5%) | - |
| **C-criteria "marginal ledge"** | | | | | | | | | | |
|  | | | enamel/composite margin | | | | dentin/composite margin | | | |
|  |  |  | code 0 | code 1 | code 2 | code 3 | code 0 | code 1 | code 2 | code 3 |
| examination time | group | number of fillings examined | no ledge | positive ledge | negative ledge | positive and negative ledge | no ledge | positive ledge | negative ledge | positive and negative ledge |
| baseline | test group 1 | n=21 | 21 (100.0%) | - | - | - | 100.0 | - | - | - |
|  | test group 2 | n=21 | 21 (100.0%) | - | - | - | 100.0 | - | - | - |
|  | control | n=33 | 33 (100.0%) | - | - | - | 100.0  ,0 | - | - | - |
| 6 months | test group 1 | n=18 | 15 (83.3%) | 1 (5.6%) | 2 (11.1%) | - | 14 (77.8%) | - | 4 (22.2%) | - |
|  | test group 2 | n=18 | 14 (77.8%) | 4 (22.2%) | - | - | 15 (83.3%) | 2 (11.1%) | 1 (5.6%) | - |
|  | control | n=28 | 15 (53.6%) | 12 (42.9%) | 1 (3.6%) | - | 21 (75.0%) | 4 (14.3%) | 3 (10.7%) | - |
| 12 months | test group 1 | n=15 | 10 (66.7%) | 4 (26.7%) | 1 (6.7%) | - | 11 (73.3%) | - | 4 (26.7%) | - |
|  | test group 2 | n=16 | 10 (62.5%) | 6 (37.5%) | - | - | 9 (56.3%) | 4 (25.0%) | 2 (12.5%) | 1 (6.3%) |
|  | control | n=24 | 5 (20.8%) | 15 (62.5%) | 4 (16.7%) | - | 14 (58.4%) | 2 (8.3%) | 6 (25.0%) | 2 (8.3%) |
| 24 months | test group 1 | n=14 | 8 (57.1%) | 5 (35.7%) | 1 (7.1%) | - | 9 (64.3%) | - | 5 (35.7%) | - |
|  | test group 2 | n=15 | 7 (46.7%) | 7 (46.7%) | 1 (6.7%) | - | 8 (53.3%) | 3 (20.0%) | 4 (26.7%) | - |
|  | control | n=21 | 3 (14.3%) | 14 (66.7%) | 3 (14.3%) | 1 (4.8%) | 16 (76.2%) | 2 (9.5%) | 3 (14.3%) | - |
| **C-criteria "marginal discoloration"** | | | | | | | | | | |
|  | | | enamel/composite margin | | | | dentin/composite margin | | | |
|  |  |  | code 0 | code 1 | code 2 | code 3 | code 0 | code 1 | code 2 | code 3 |
| examination time | group | number of fillings examined | no marginal discoloration | discoloration  on up to 1/3 of the circumference | discoloration  on more than 1/3 of the circumference | secondary caries with cavitation | no marginal discoloration | discoloration  on up to 1/3 of the circumference | discoloration  on more than 1/3 of the circumference | secondary caries with cavitation |
| baseline | test group 1 | n=21 | 21 (100.0%) | - | - | - | 21 (100.0%) | - | - | - |
|  | test group 2 | n=21 | 21 (100.0%) | - | - | - | 21 (100.0%) | - | - | - |
|  | control | n=33 | 33 (100.0%) | - | - | - | 33 (100.0%) | - | - | - |
| 6 months | test group 1 | n=18 | 17 (94.4%) | 1 (5.6%) | - | - | 18 (100.0%) | - | - | - |
|  | test group 2 | n=18 | 15 (83.3%) | 3 (16.7%) | - | - | 18 (100.0%) | - | - | - |
|  | control | n=29 | 21 (72.7%) | 8 (27.6%) | - | - | 29 (100.0%) | - | - | - |
| 12 months | test group 1 | n=15 | 13 (86.7%) | 1 (6.7%) | 1 (6.7%) | - | 14 (93.3%) | 1 (6.7%) | - | - |
|  | test group 2 | n=16 | 11 (68.8%) | 5 (31.3%) | - | - | 14 (87.5%) | 1 (6.3%) | 1 (6.3%) | - |
|  | control | n=24 | 14 (58.3%) | 8 (33.3%) | 2 (8.3%) | - | 23 (95.8%) | 1 (4.2%) | - | - |
| 24 months | test group 1 | n=14 | 11 (78.6%) | 1 (7.1%) | 2 (14.3%) | - | 12 (85.7%) | 2 (14.3%) | - | - |
|  | test group 2 | n=15 | 5 (33.3%) | 10 (66.7%) | - | - | 12 (80.0%) | 1 (6.7%) | 2 (13.3%) | - |
|  | control | n=22 | 11 (50.0%) | 8 (36.4%) | 3 (13.6%) | - | 21 (95.5%) | 1 (4.5%) | - | - |
